# Using magnetic mesoporous silica nanoparticles armed with EpCAM aptamer as an efficient platform for specific delivery of 5-fluorouracil to colorectal cancer cells

**DOI:** 10.3389/fbioe.2022.1095837

**Published:** 2023-01-06

**Authors:** Aseel Kamil Mohammad Al-Mosawi, Ahmad Reza Bahrami, Sirous Nekooei, Amir Sh. Saljooghi, Maryam M. Matin

**Affiliations:** ^1^ Department of Biology, Faculty of Science, Ferdowsi University of Mashhad, Mashhad, Iran; ^2^ Industrial Biotechnology Research Group, Institute of Biotechnology, Ferdowsi University of Mashhad, Mashhad, Iran; ^3^ Department of Radiology, Qaem Hospital, Mashhad University of Medical Sciences, Mashhad, Iran; ^4^ Department of Chemistry, Faculty of Science, Ferdowsi University of Mashhad, Mashhad, Iran; ^5^ Novel Diagnostics and Therapeutics Research Group, Institute of Biotechnology, Ferdowsi University of Mashhad, Mashhad, Iran; ^6^ Stem Cells and Regenerative Medicine Research Group, Academic Center for Education Culture and Research (ACECR), Mashhad, Iran

**Keywords:** colorectal cancer, mesoporous silica nanoparticles, superparamagnetic iron oxide, 5-fluorouracil, epithelial cell adhesion molecule

## Abstract

**Background:** Theranostic nanoparticles with both imaging and therapeutic capacities are highly promising in successful diagnosis and treatment of advanced cancers.

**Methods:** Here, we developed magnetic mesoporous silica nanoparticles (MSNs) loaded with 5-fluorouracil (5-FU) and surface-decorated with polyethylene glycol (PEG), and epithelial cell adhesion molecule (EpCAM) aptamer (Apt) for controlled release of 5-FU and targeted treatment of colorectal cancer (CRC) both *in vitro* and *in vivo*. In this system, Au NPs are conjugated onto the exterior surface of MSNs as a gatekeeper for intelligent release of the anti-cancer drug at acidic conditions.

**Results:** Nanocarriers were prepared with a final size diameter of 78 nm, the surface area and pore size of SPION-MSNs were calculated as 636 m^2^g^−1^, and 3 nm based on the BET analysis. The release of 5-FU from nanocarriers was pH-dependent, with an initial rapid release (within 6 h) followed by a sustained release for 96 h at pH 5.4. Tracking the cellular uptake by flow cytometry technique illustrated more efficient and higher uptake of targeted nanocarriers in HT-29 cells compared with non-targeted formula. *In vitro* results demonstrated that nanocarriers inhibited the growth of cancer cells *via* apoptosis induction. Furthermore, the targeted NPs could significantly reduce tumor growth in immunocompromised C57BL/6 mice bearing HT-29 tumors, similar to those injected with free 5-FU, while inducing less side effects.

**Conclusion:** These findings suggest that application of Apt-PEG-Au-NPs@5-FU represents a promising theranostic platform for EpCAM-positive CRC cells, although further experiments are required before it can be practiced in the clinic.

## 1 Introduction

Colorectal cancer (CRC) is a heterogeneous disease, which ranks as the third most lethal malignancy in the world (Marquez et al., 2018). A wide variety of chemotherapeutic drugs have been applied for CRC treatment, one of which is 5-fluorouracil (5-FU) as the first-line conventional chemotherapeutic compound in the clinic that can be employed either as monotherapy or in combination with other treatments (Chakrabarti et al., 2019). The major challenges in clinical application of 5-FU include lack of specificity, drug resistance, damage to normal cells and low patient survival rates (Sethy and Kundu, 2021). Thus, numerous studies are being performed in order to discover novel therapeutic strategies for CRC therapy. Recently, use of carrier mediated drug delivery systems (DDSs) offers multiple benefits to overcome adverse and non-specific side effects of free chemotherapeutic agents (Dang and Guan, 2020; Er et al., 2020). Superparamagnetic iron oxide nanoparticles (SPIONs) conjugated with mesoporous silica nanoparticles (MSNs) as a well-known inorganic platform can be applied for DDSs. Generally, SPIONs formed by crystals of iron oxide (magnetite Fe_3_O_4_ or maghemite γ-Fe_2_O_3_) with remarkable physicochemical characteristics such as being non-toxic, and non-immunogenic, having contrast properties, and easy modification, have gained a lot of interest in the field of cancer therapy (Palanisamy and Wang, 2019). Moreover, SPIONs can be coated with various biocompatible polymers in order to improve their colloidal stability and avoid unwanted iron release (Iranpour et al., 2021a). In this regard, MSNs are one of the excellent candidates due to their porous structure, big surface area, good biocompatibility, easy surface functionalization, low toxicity, controllable drug release and low cost (Yang et al., 2019; Kankala et al., 2020a; Kundu et al., 2020; Spoială et al., 2021). Encapsulating anti-cancer agents in pore tunnels capped with fabricated controlled gatekeepers would lead to avoiding drug leakage during delivery process (Wen et al., 2017). Intelligent drug release behavior can be achieved by internal and external stimuli causing uncapping, alteration of the MSNs structure and improved drug delivery performance at the target locations (Abdo et al., 2020; Kankala et al., 2022). In this context, Au nanoparticle gatekeepers have been covalently conjugated onto the pore entrance of MSNs; they did not allow the release of guest molecules unless under endogenous acidic triggers (Cui et al., 2014; Babaei et al., 2017). Active targeting strategy is one of the promising approaches that involves ligand-mediated targeting for delivery of nanocarriers to specific locations. Up to now wide variety of ligands such as peptides, antibodies, polysaccharides and aptamers have been employed as targeting moieties. Among them, aptamers as small strands of DNA or RNA have been commonly considered as suitable targeting agents due to their stability, high binding affinity, relatively low cost and little or no immunogenicity in comparison with other targeting ligands (Navale et al., 2015; Dhar et al., 2020). Epithelial cell adhesion molecule (EpCAM) is a transmembrane glycoprotein, which plays critical roles in cellular signaling, cell migration, proliferation, and differentiation (Gires et al., 2020). It was demonstrated that, expression of this marker is associated with CRC development and metastasis and therefore, it can be considered as a good therapeutic target for CRC treatment (Songun et al., 2005; Mokhtari and Zakerzade, 2017).

In this study, we developed non-targeted and targeted PEGylated SPION-MSNs for controlled release of 5-FU. In this system, Au NPs are conjugated onto the exterior surface of MSNs as gatekeepers for intelligent release of anti-cancer drugs at acidic condition. Considering differential EpCAM expression in cancerous and normal cells, we evaluated the anti-cancer capability of prepared nanocarriers against both EpCAM positive and negative cell lines *in vitro*. In the next stage, *in vivo* (tumor growth rate, body weight loss, biodistribution and side effects) performance of nanocarriers was evaluated in immunocompromised C57BL/6 mice bearing human colorectal tumors.

## 2 Experimental section

### 2.1 Materials

All chemical reagents and solvents were purchased from Merck and used as received without further purification. SPION, ammonium hydroxide (NH_4_OH), 3-aminopropyltriethoxysilane (APTES), tetraethyl orthosilicate (TEOS), tetramethylammonium hydroxide, cetyltrimethylammonium bromide (CTAB), 4’, 6-diamidino-2-phenylindole (DAPI), and Rhodamine B were purchased from Sigma-Aldrich Co. (Germany). Heterofunctional polyethylene glycol (PEG) polymer with a terminal thiol and carboxylic acid functional groups (SH–PEG–COOH, Mw: 3,500) was purchased from JenKem (United States of America). 3-(4, 5-dimethylthiazol-2-yl)-2, 5-diphenyltetrazolium bromide (MTT) and trypsin-EDTA were obtained from Tinab Shimi (Iran). Matrigel^®^ matrix (DLW354263) was purchased from Corning Inc. (United States of America). Fetal bovine serum (FBS), penicillin-streptomycin, Roswell Park Memorial Institute 1640 (RPMI 1640) medium and Dulbecco’s modified Eagle’s medium (DMEM) were obtained from Gibco (Scotland). The 5′-amine-anti-EpCAM DNA aptamer (5′-CAC​TAC​AGA​GGT​TGC​GTC​TGT​CCC​ACG​TTG​TCA​TGG​GGG​GTT​GGC​CTG-3′) was custom synthesized by MicroSynth (Balgach, Switzerland (Song et al., 2013). Fluorescein isothiocyanate (FITC) annexin V apoptosis detection kit with propidium iodide was bought from BioLegend (United States of America).

### 2.2 Synthesis procedure

**FIGURE 1:**
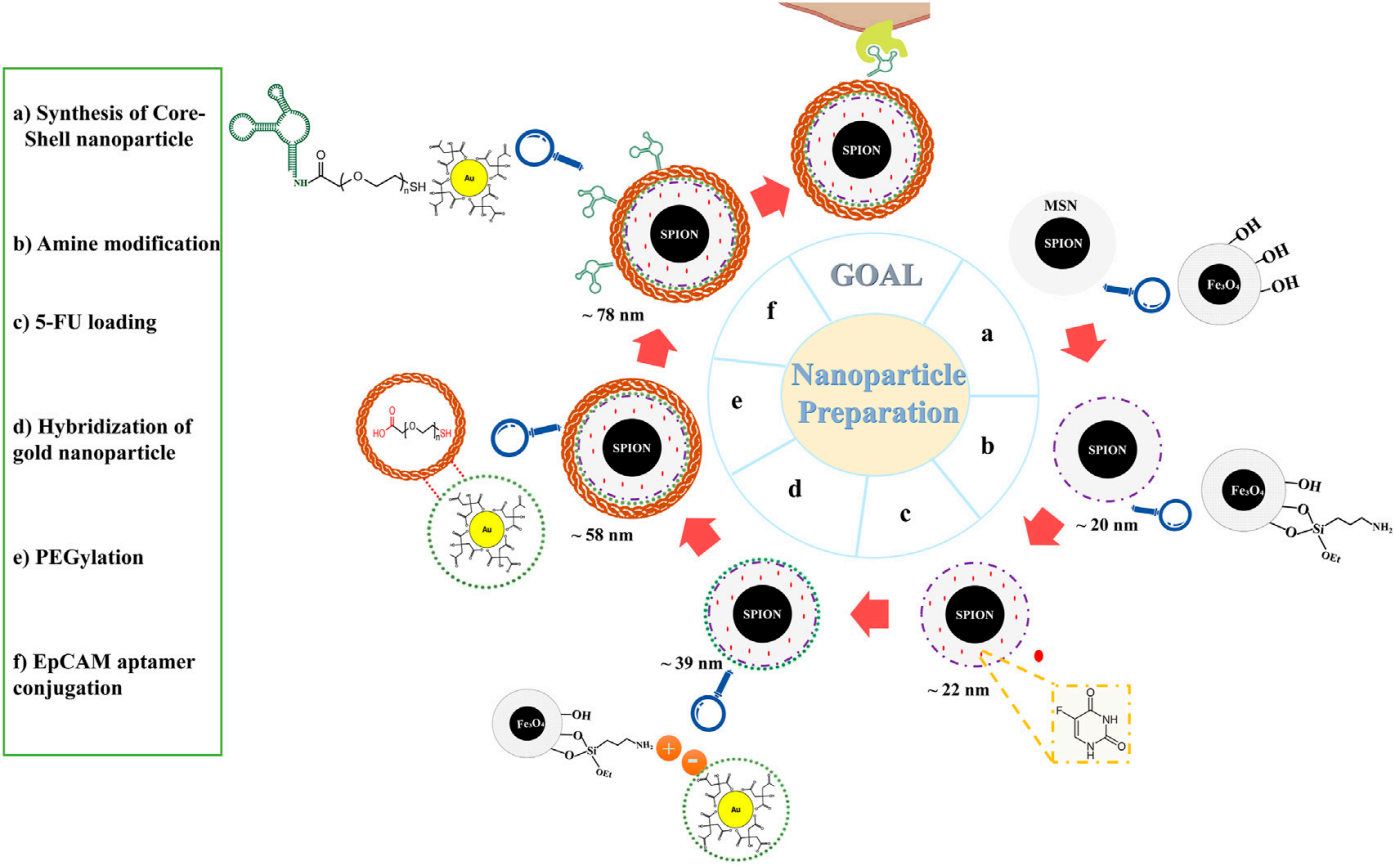
Schematic illustration of nanoparticles preparation. Abbreviations:*SPION* Superparamagnetic iron oxide nanoparticle, *MSN* Mesoporous silica nanoparticle, *5-FU* 5-Fluorouracil, *PEG* Polyethylene glycol, *EpCAM* Epithelial cell adhesion molecule.

For better understanding, a schematic diagram of the designed functional NPs is shown in Figure 1.

#### 2.2.1 Synthesis of core-shell SPION-MSNs

0.2 g of SPIONs were dispersed in 10 ml distilled water and ethanol under vigorous stirring for 30 min, then 1 ml aqueous TEOS was added slowly. The reaction was conducted for 2 h under nitrogen atmosphere with severely stirring at 40°C. In the next step, SPION-MSNs were separated with an external magnet and dissolved in 0.75 g CTAB, and 1 ml ammonium hydroxide (25%). After stirring for 2 h at 60°C, the purified product was collected by centrifugation at 6,000 *g* for 15 min and repeatedly washed with ethanol/water mixture. Core-shell prepared SPION-MSNs were dried (according to classical template) at 550°C for 5 h in order to perform calcination (Zhang et al., 2010; Avedian et al., 2018).

#### 2.2.2 Synthesis of NH_2_-modified SPION-MSNs

SPION-MSNs were functionalized with APTES to obtain aminated nanocarriers. 320 mg of the previous step products were dispersed in 100 ml ethanol and 1,200 µl APTES under vigorous stirring at room temperature. After 24 h, the SPION-MSNs-NH_2_ were collected by centrifuging the mixture at 10,000 *g* for 20 min to remove excess APTES, washed with ethanol three times and dried in vacuum overnight.

#### 2.2.3 Loading 5-FU/Rhodamine B into the SPION-MSNs-NH_2_

Typically, 2 mg of the SPION-MSNs-NH_2_ sample were suspended in 1 ml 5-FU or Rhodamine B (2 mg/ml) solution, sonicated for 20 min, and stirred for 48 h at room temperature. To evaluate the encapsulation content, the suspension was centrifuged (17,000 *g* for 20 min) and the amounts of free 5-FU or Rhodamine B in supernatant were determined by (UV-Vis; Eppendorf, Germany) at 265 and 543 nm, respectively. Loading capacity (LC) and encapsulation efficiency (EE) were assessed indirectly according to the following Eq. 1:

$$\begin{array}{c} EE\%=\frac{Initial\;amount\;of\;5‐FU\;added-amount\;of\;free\;5‐FU\;in\;supernatant}{Initial\;amount\;of\;5‐FU\;added}\times 100\\ LC\%=\frac{Initial\;amount\;of\;5‐FU-amount\;of\;free\;5‐FU\;in\;supernatant}{Mass\;of\;final\;formulation}\times 100 \end{array}$$ (1)

#### 2.2.4 Synthesis of gold gatekeeper and capped SPION-MSNs@5-FU

100 ml aqueous solution containing 0.21 mM HAuCl_4_ and 2.5 mM trisodium citrate was first prepared. Then, 5 ml of ice-cold fresh 0.1 M NaBH_4_ was added to the solution while vigorously rousing. The solution turned pink immediately after adding NaBH_4_, demonstrating the formation of gold nanoparticles (Iranpour et al., 2021b). In the next step, 1 ml of prepared gold NPs were added to 2 mg/ml of previous step products and stirred for 24 h at room temperature. The final product at this stage was abbreviated as Au-NPs@5-FU.

#### 2.2.5 Synthesis of PEG-Au-NPs@5-FU & Apt-PEG-Au-NPs@5-FU

6 mg of the bifunctional PEG (SH–PEG–COOH) was added to the pervious suspension and further stirred for 24 h at room temperature. In the next step, the amine head group of EpCAM aptamer was covalently attached to carboxylic group of PEG *via* EDC/NHS as activating agents. For this aim, EDC (4 mg) and NHS (3 mg) were added to the suspension of PEG-Au-NPs@5-FU and stirred for 30 min to activate the carboxylic acid groups. Then working solution of EpCAM aptamer (20 μl, 5 μM) was added to the suspension and stirred overnight at 4°C. Finally, target nanocarriers were achieved by centrifugation at 17,000 *g* for 20 min and washed three times with DNase/RNase‐free distilled water.

### 2.3 Physicochemical characterizations

Fourier transform infrared (FTIR) spectra of all samples were evaluated by pressed KBr pellets using an AVATAR 370 FTIR spectrometer (Therma Nicolet spectrometer, United States of America). Particle size and zeta potential of nanocarriers in each step were determined by dynamic light scattering (DLS) using a Malvern Zeta sizer Nano ZS90 (Malvern Instruments, Worcestershire, United Kingdom) at 258°C. Morphology, size, shape, and homogeneity of SPION-MSNs were monitored by field emission scanning electron microscopy (FESEM; TESCAN MIRA, Czech Republic) and high resolution-transmission electron microscopy (HR-TEM; FEI, United States of America). Surface areas and pore size distributions of SPION@MSNs and Au-NPs@5-FU were calculated using the Brunauer-Emmett-Teller (BET) and Barrett-Joyner-Halenda (BJH) methods, respectively. Elemental compositions (C, O, N, Si, Fe, and Au) were determined with the energy-dispersive X-ray spectroscopy (EDX; TESCAN MIRA, Czech Republic). The magnetic behavior of NPs and PEG-Au-NPs@5-FU was also estimated using a vibrating-sample magnetometer (VSM, LakeShore 7,400). Thermogravimetric analysis (TGA) was achieved over the temperature range 25 –880°C at a heating rate of 20 °C min^−1^ under N_2_ atmosphere with a Shimadzu thermogravimetric analyzer (TGA; TA, United States of America). Conjugation of the EpCAM aptamer on the surface of nanocarriers was determined by agarose gel electrophoresis. All samples (DNA ladder, PEG-Au-NPs@5-FU, and Apt-PEG-Au-NPs@5-FU) were loaded onto a 2% agarose gel prepared in Tris-borate-EDTA (TBE) solution (1X). After electrophoresis (85 V, 40 min), the gel was stained with ethidium bromide and photographed using a gel documentation system (Major Science, Taiwan).

### 2.4 *In vitro* drug release measurements

The release evaluation of 5-FU was performed in buffer solutions with two different pH values (pH 7.4 and 5.4) using dialysis membrane (cut off = 1,000). Drug-loaded Au-NPs (3 mg) in sealed dialysis membrane were placed in a larger vessel containing 30 ml release medium in a shaker incubator set at 37°C and 100 rpm. Sampling was done (3 ml) at specific time points and substituted with 3 ml of fresh medium to keep the volume constant. Finally, the samples were investigated by UV-Vis spectroscopy at 265 nm. All assays were carried out in triplicate.

### 2.5 *In vitro* cytotoxicity assay

Human colorectal adenocarcinoma HT-29 cells and mouse fibroblast NIH/3T3 cells were obtained from the National Cell Bank of Iran (Pasteur Institute, Tehran) and cultured in RPMI 1640 and DMEM medium, respectively, supplemented with 10% FBS, penicillin (100 U/mL) and streptomycin (100 μg/ml) in a humidified incubator with 5% CO_2_ at 37°C.

Cytotoxicity of free 5-FU, non-targeted, and targeted nanocarriers were assessed on HT-29 and NIH/3T3 cells using the MTT assay. For this aim, both cell lines were treated with different concentrations of mentioned compounds (3.125–100 μg/ml) in triplicate. After 24, 48 and 72 h, 20 µl of MTT solution (5 mg/ml in PBS) was added to each well and incubated for 4 h at 37°C. Afterwards, the media were replaced with 150 µl DMSO to dissolve the insoluble formazan crystals. Finally, the optical density (OD) was measured at 540 nm using a microplate reader (Awareness Technology, United States of America). Cell viability was measured using the Eq. 2 and the IC_50_ values of compounds were calculated from the semi-logarithmic dose-response curves using GraphPad Prism 6.01.

$$Cell\;viability\%=\frac{OD\;of\;samples-OD\;of\;blank}{OD\;of\;control-OD\;of\;blank}\times 100$$ (2)

### 2.6 Hemolysis assay

Normal human blood from a healthy volunteer donor was used to test the hemolysis effects of NPs. The red blood cells (RBCs) were collected with cold centrifugation at 1,500 *g* for 10 min. Following repeated washing, the pellet was rinsed five times with PBS (pH 7.4). The diluted RBC suspensions were incubated with equal volume of SPION-MSNs or PEG-Au@NPs-5-FU with different concentrations (200, 100, 50, 25 and 12.5 mg/ml) for 12 and 24 h at 37°C. To obtain 0% and 100% hemolysis, PBS and water were added in equal volumes to the RBC suspensions, respectively. The mixtures were then centrifuged at 2,500 *g* for 1 min and the supernatants were analyzed for presence of hemoglobin at 540 nm using a microplate reader (Awareness Technology, United States of America). The degree of hemolysis was measured using the following Equation (3) (Lin and Haynes, 2010; Zhao et al., 2011):

$$Hemolysis\%=\frac{OD\;of\;sample-OD\;of\;negative\;control}{OD\;of\;positive\;control-OD\;of\;negative\;control}\times 100$$ (3)

### 2.7 *In vitro* cellular uptake assessment

Intracellular uptake of targeted and non-targeted nanocarriers was evaluated by both flow cytometry and fluorescence microscopy. For this purpose, HT-29 and NIH/3T3 cells were seeded in six-well plates at a density of 2 × 10^5^ cells/well and incubated overnight for attachment in 5% CO_2_ atmosphere at 37°C. Then, either non-targeted or targeted Rhodamine B-loaded nanocarriers at equal 5-FU concentration (5 μg/ml) were added to each well.

For flow cytometry analysis, the media were removed and cells were washed twice with 1 ml cold PBS (1X), trypsinized and centrifuged at 400 *g* for 5 min. The cell pellets were resuspended in 200 µl cold PBS and the intensity of Rhodamine B fluorescence was then determined using the BD Accuri C6, equipped with 488 nm laser detector in the FL2 channel. The results were analyzed using FlowJo 7.6.1 software. Additionally, the cellular uptake was further studied by fluorescence microscopy. In this context, both cell types were washed with cold PBS and fixed in 4% v/v paraformaldehyde for 20 min at 4°C. After staining fixed cells with DAPI fluorescent dye, samples were viewed under the Nikon E1000M fluorescent microscope (Nikon, Tokyo, Japan).

### 2.8 Investigating cell death mechanism

Flow cytometry analysis was performed to investigate the mechanism of cell death induced by nanocarriers. HT-29 and NIH/3T3 cells as EpCAM positive and negative surface marker, respectively, were seeded into six-well plates and treated with targeted and non-targeted nanocarriers at equal to 5-FU concentrations (5 μg/ml). Then, FITC/annexin V apoptosis detection kit was used based on the manufacturer’s protocol to stain both treated cell types with propidium iodide (PI) and annexin V. Subsequently, the samples were evaluated using the BD Accuri C6 flow cytometer with FL1 and FL2 filters and results were analyzed *via* FlowJo 7.6.1 software.

### 2.9 *In vivo* studies

#### 2.9.1 Investigating anti-cancer properties of nanocarriers

The animal experiments were approved and conducted in accordance with Animal Ethics Committee of Ferdowsi University of Mashhad (IR.UM.REC.1400.013). Immunosuppression of 4–6 weeks old male C57BL/6 mice was performed based on our previous report (Iranpour et al., 2021c). Afterwards, HT-29 cells with a concentration of 8 × 10^6^ cells per 150 μl mixture of FBS:Matrigel (1:1, v/v) were subcutaneously injected into the right flank of each immunocompromised animal. When the tumor volume reached 100–150 mm^3^, the mice were randomly divided into four different experimental groups (5 mice per group) including: control (PBS treatment), non-targeted nanocarriers, targeted nanocarriers, and free 5-FU group. The injections were performed (2 mg/kg body weight) intravenously *via* the tail vein for 4 times on days 0, 3, 6 and 9. Tumor volume was measured using the Eq. 4:

$$Tumor\;volume=\frac{height\times length\times width}{2}$$ (4)

Tumor volume and body weight were measured every day for up to 15 days to evaluate the anti-tumor activity of nanocarriers and free 5-FU. To investigate *in vivo* toxicity and possible side effects, mice were sacrificed at the end of the 15th day, and major organs (kidney, liver, heart, spleen, and lung) were collected and fixed in 10% paraformaldehyde solution. Then, the fixed tissues were sectioned and stained with hematoxylin and eosin (H&E) for histological examination using standard techniques. The slides were observed and photographed using an optical microscope (Olympus IX70; Japan) equipped with a camera. Meanwhile, the distribution of both targeted and non-targeted nanocarriers was explored 12 and 24 h post injection using an *in vivo* imaging system (IVIS, Xenogen, CA; 100 series).

#### 2.9.2 *In vivo* MR imaging

The non-targeted and targeted nanocarriers were intravenously injected to the lateral tail vein of immunocompromised C57BL/6 mice bearing human HT-29 tumors. 12 and 24 h post injection, MRI was performed under a 1.5 T MRI scanner (Magnetom Symphony; Siemens, Germany) with following parameters: protocol = turbo spin echo (TSE), repetition time (TR) = 5000 ms, echo time (TE) = 91 ms, resolution = 384 × 384 pixel, and slice thickness = 3 mm to investigate the intratumoral accumulation of targeted and non-targeted conjugates.

#### 2.9.3 Statistical analysis

Statistical analysis was performed using GraphPad Prism version 6.01 (GraphPad software, San Diego, CA). Results are expressed as mean ± SD or SEM. *p* < 0.05 was considered to be statistically significant.

## 3 Results

### 3.1 Synthesis and characterization of nanoparticles

**FIGURE 2:**
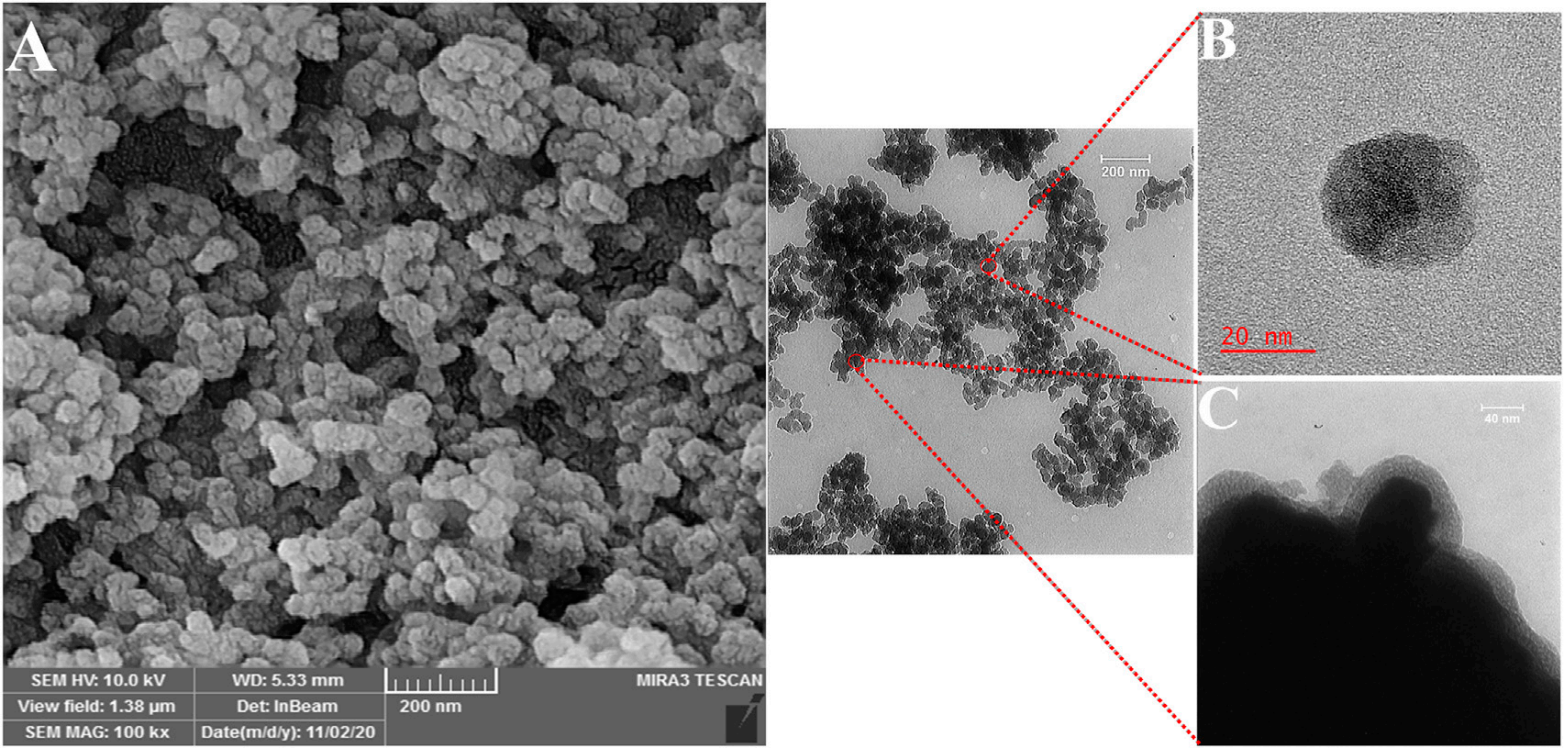
Morphological characterization of the magnetic mesoporous silica nanoparticles (SPION-MSNs). **(A)** Scanning electron microscopy, **(B)** and **(C)** High resolution-transmission electron microscopy. Abbreviations: *SPION* Superparamagnetic iron oxide nanoparticle and *MSN* Mesoporous silica nanoparticle.

**FIGURE 3:**
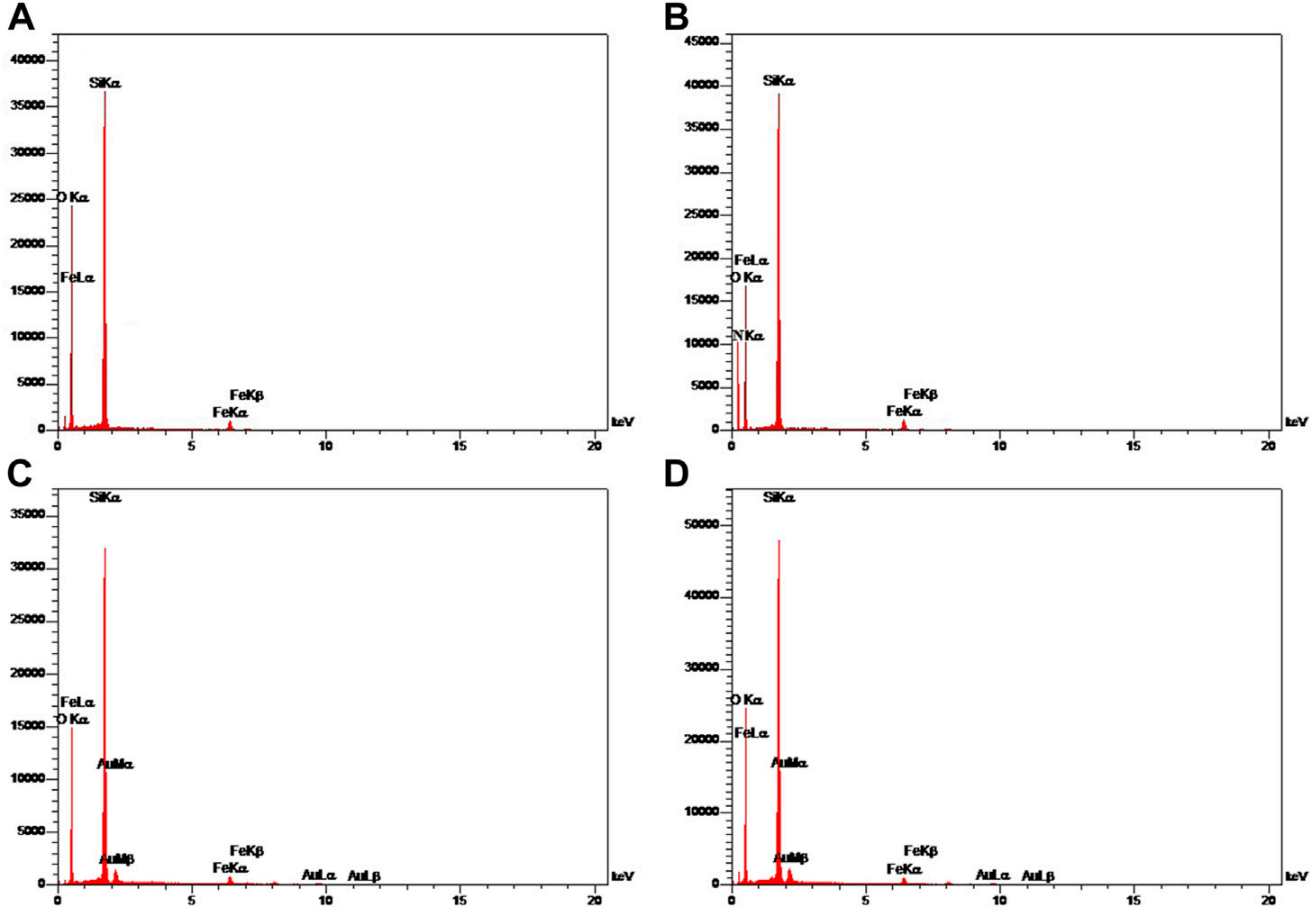
Energy-dispersive X-ray (EDX) mapping of **(A)** SPION-MSNs, **(B)** SPION-MSNs-NH_2_ **(C)** Au-NPs@5-FU and **(D)** PEG-Au-NPs@5-FU. Abbreviations: *SPION* Superparamagnetic iron oxide nanoparticle, *MSN* Mesoporous silica nanoparticle, *5-FU* 5-Fluorouracil, *PEG* Polyethylene glycol.

**FIGURE 4:**
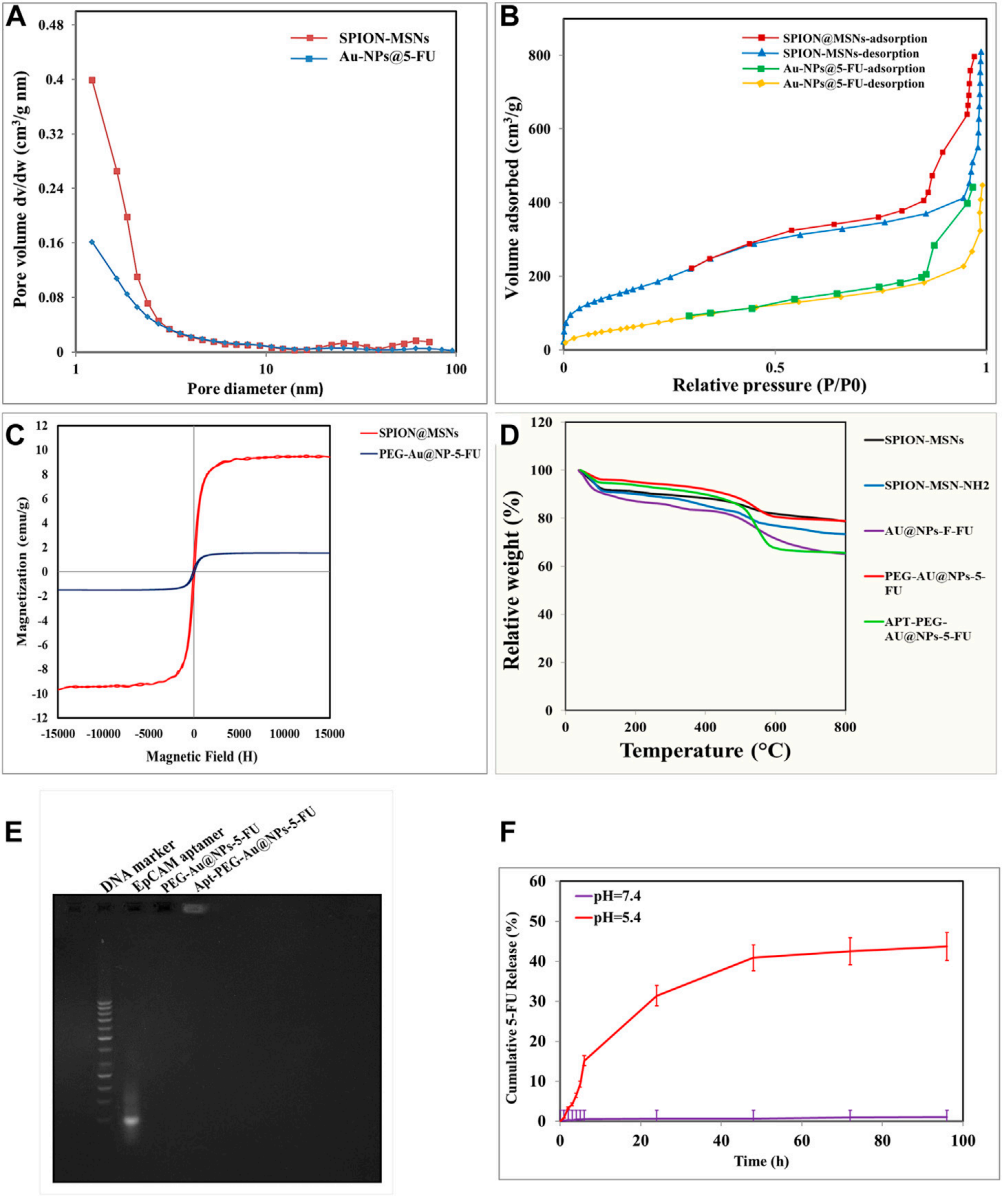
Characterization of prepared nanoparticles by various techniques. **(A)** BJH pore size distribution. **(B)** BET N_2_ adsorption/desorption curves of SPION-MSNs and Au-NPs@5-FU. **(C)** Vibrating-sample magnetometer (VSM) of SPION-MSNs and PEG-Au-NPs@5-FU. **(D)** Thermogravimetric analysis (TGA) curves of SPION-MSNs, SPION-MSNs-NH_2_, Au-NPs@5-FU, PEG-Au-NPs@5-FU and Apt-PEG-Au-NPs@5-FU. **(E)** Confirmation of aptamer bioconjugate by gel electrophoresis. **(F)** 5-FU release profile from nanocarriers in phosphate‐buffered saline (PBS) (pH 7.4) and citrate buffer (pH 5.4). Abbreviations: *SPION* Superparamagnetic iron oxide nanoparticle, *MSN* Mesoporous silica nanoparticle, *NP* nanoparticle, 5-FU 5-Fluorouracil, *VSM* Vibrating-sample magnetometer, *PEG* Polyethylene glycol, *TGA* Thermogravimetric analysis, *Apt* Aptamer, *EpCAM* Epithelial cell adhesion molecule.

The structure and magnetic properties of the prepared nanocarriers were fully characterized by diverse techniques including FTIR, zeta potential, SEM, HR-TEM, BET, EDX, VSM, and TGA. The FTIR spectra were recorded at room temperature in the range between 4000 cm^−1^ and 400 cm^−1^ and the results are presented in Supplementary Figure S1. The additional transmittance peaks were observed in each step of modification at the surface of nanocarriers. Furthermore, zeta potential measurements were used to explore the surface charge of nanocarriers and results of this analysis as shown in Table 1 indicated that the synthesized formulations had both negative and positive charges with values ranging from −10 to +14 mV. The hydrodynamic diameters were also obtained from DLS measurements ranging from 20 to 78 nm (Table 1). The morphology of SPION-MSNs was investigated by SEM and HR-TEM techniques. MSNs were fabricated with the diameter of about 31 nm, according to SEM results (Figure 2A). The average particle size of SPION-MSNs obtained by HR-TEM analysis was lower than that of DLS analysis and clearly indicated the contrast core-shell structure of the nanocarriers (Figure 2C). To further study the element distribution, the chemical purity of the samples was verified by EDX. The EDX analysis of nanocarriers is indicated in Figure 3, which shows the existence of C, O, N, Si, Fe and, Au in the structure of nanocarriers and confirms successful fabrication of different nanocarrier platforms. Additionally, the pore size (Figure 4A) and surface area (Figure 4B) of SPION-MSNs were calculated as 3 nm, and 636 m^2^g^−1^ based on the BJH and BET analysis of the N_2_ sorption isotherm, respectively. The fact that BET surface area of SPION-MSNs became smaller after the loading of 5-FU and reached to 269 m^2^g^−1^, indicates that anti-cancer drug has been able to fill the channels of the nanocarriers. Moreover, EE% and LC% were measured as 98% and 49%, respectively. Afterwards, the VSM analysis was performed to compare the magnetic property of nanocarriers after PEGylation (Figure 4C). As it is evident from the resulting magnetization curves, the saturation magnetization amount of SPION-MSNs and PEG-Au-NPs@5-FU are −9 and −2 emu g^−1^, respectively (Figure 4C). For quantitative analysis, TGA was further performed as shown in Figure 4D; results indicated that the weight loss of SPION-MSNs was 21.54%, while the weight loss of Apt-PEG-Au-NPs@5-FU increased to 34.56% in the same temperature range. Furthermore, the conjugation of EpCAM aptamer with PEG-Au-NPs@5-FU was monitored by agarose gel electrophoresis. As shown in Figure 4E, the migration of free EpCAM Apt matched that of the 50 base pair size marker, while Apt-PEG-Au-NPs@5-FU mainly remained at the origin. Taken together, these data revealed the efficient conjugation between PEG-Au-NPs@5-FU and EpCAM aptamer.

**TABLE 1:** Average values of size, PDI, and zeta potential of different formulations synthesized in this study.

Sample	Size (nm)	Polydispersity index (PDI)	Zeta (mV)
SPION-MSN-NH_2_	20.3 ± 2.7	0.4 ± 0.02	+14 ± 5.7
NPs@5-FU	21.8 ± 1.5	0.1 ± 0.63	+7 ± 2.3
Au-NPs@5-FU	38.9 ± 1.0	0.2 ± 0.02	−14 ± 1.1
`PEG-Au-NPs@5-FU	58.2 ± 3.3	0.3 ± 0.14	−11 ± 1.5
Apt-PEG-Au-NPs@5-FU	78.1 ± 2.1	0.2 ± 0.11	−10 ± 0.8

Abbreviations: *SPION*, superparamagnetic iron oxide nanoparticle; *MSN*, mesoporous silica nanoparticle; *5-FU*, 5-Fluorouracil; *NP*, nanoparticle; *PEG*, polyethylene glycol; *Apt*, Aptamer Data are expressed as mean ± SD.

### 3.2 pH Dependent drug release

*In vitro* drug release behaviors of 5-FU encapsulated nanocarriers proposed labile behavior of synthetic compounds under two different pH values*.* The 5-FU release from Au-NPs@5-FU was investigated at different time intervals and various pH values (5.4 and 7.4). As evident in Figure 4F, the release of 5-FU from nanocarriers was pH-dependent, with an initial rapid release (within 6 h) followed by a sustained release for 96 h at pH 5.4. The sustained release of 5-FU demonstrated that SPION-MSNs might improve anti-cancer drugs efficacy and reduce related toxicities.

### 3.3 Specific targeting of Apt-PEG-Au-NPs@5-FU *in vitro*

**FIGURE 5:**
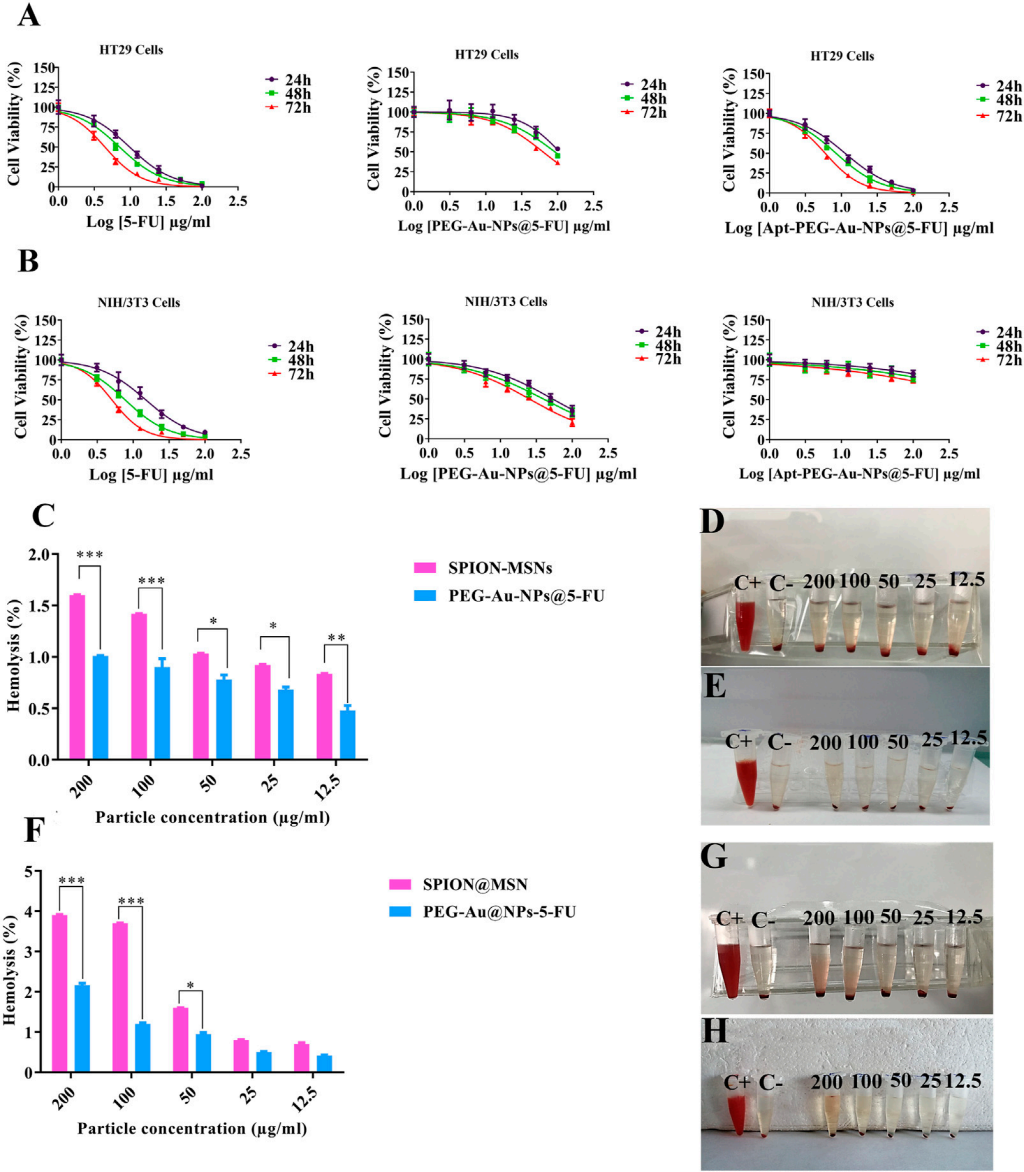
Cytotoxicity of nanocarriers as evaluated by MTT assay. Dose-response curves of free 5-FU, PEG-Au-NPs@5-FU and Apt-PEG-Au-NPs@5-FU formulations against **(A)** HT-29 and **(B)** NIH/3T3 cells at 24, 48 and 72 h **(C)** Hemolysis percentage after 12 h and **(F)** after 24 h incubation of red blood cells with the SPION-MSNs and PEG-Au-NPs@5-FU. Data represent the mean ± SD of three independent experiments, *p < 0.05, **p < 0.01, ***p < 0.001. Visual inspections of the tubes containing diluted blood samples after exposure to different concentrations of **(D,G)** SPION-MSNs and **(E,H)** PEG-Au-NPs@5-FU for 12 and 24 h are presented on the right panel. Abbreviations: *SPION* Superparamagnetic iron oxide nanoparticle, *MSN* Mesoporous silica nanoparticle, *PEG* Polyethylene glycol, *NP* nanoparticle, *Apt* Aptamer, *5-FU* 5-Fluorouracil.

To evaluate the effectiveness of aptamer in targeting EpCAM cell surface protein and transporting 5-FU into the cells, anti-cancer activity of PEG-Au-NPs@5-FU, Apt-PEG-Au-NPs@5-FU and free 5-FU at different concentrations of 5-FU was evaluated against 2 cell lines including HT-29 (EpCAM^+^) and NIH/3T3 (EpCAM^−^) using MTT assay (Figure 5). Cytotoxicity of the backbone, SPION@MSNs, was first evaluated on both cell lines. The results demonstrated that the prepared nanocarriers were non-toxic and observed toxicity was related to the encapsulated 5-FU (Figures 5A, B). The IC_50_ values of PEG-Au-NPs@5-FU, Apt-PEG-Au-NPs@5-FU and free 5-FU on HT-29 and NIH/3T3 cells are indicated in Table 2. In this regard, targeted nanocarrier was more toxic in comparison with non-targeted nanocarriers in HT-29 cells, demonstrating that our designed targeted formulation could specifically bind to EpCAM receptors, which are overexpressed on the surface of HT-29 cells and successfully enter the desired cells *via* receptor mediated endocytosis. Moreover, there was a considerable difference between non-targeted and targeted nanocarriers cytotoxicity on NIH/3T3 cells due to the absence of EpCAM receptor on their surface.

**TABLE 2:** IC_50_ values of free drug, non-targeted and targeted nanocarriers in HT-29 and NIH/3T3 cells at different time points.

Samples	IC_50_ (µg/ml) ± SD HT-29 cells			IC_50_ (µg/ml) ± SD NIH/3T3 cells		
	24 h	48 h	72 h	24 h	48 h	72 h
Free 5-FU	9.08 ± 1.75	7.10 ± 1.83	4.45 ± 1.85	14.98 ± 1.77	7.71 ± 1.79	5.05 ± 1.80
PEG-Au-NPs@5-FU	107.7 ± 1.81	91.61 ± 1.89	62.56 ± 1.80	53.20 ± 1.82	39.48 ± 1.82	25.23 ± 1.88
Apt-PEG-Au-NPs@5-FU	11.27 ± 1.80	8.42 ± 1.78	5.95 ± 1.78	3,007 ± 3.1	2,383 ± 5.1	1,301 ± 3.63

Abbreviations: *SPION*, superparamagnetic iron oxide nanoparticle; *Au-NPs*, gold nanoparticles; *5-FU*, 5-Fluorouracil; *PEG*, polyethylene glycol; *Apt*, Aptamer.

### 3.4 PEGylation reduces hemolysis

Hemolysis activity of SPION@MSNs and PEG-Au-NPs@5-FU samples (Figures 5C–H) indicated that the PEGylation strategy could significantly reduce RBCs hemolysis in comparison with SPION-MSNs at both 12 and 24 h. Our results demonstrated that the PEGylated nanocarriers at 200 μg/ml concentration had the highest hemolysis activity (1%) at 12 h and it was increased to 2.16% at 24 h. Furthermore, other tested concentrations (12.5–100 μg/ml) led to less than 1.2% hemolysis at both 12 and 24 h.

### 3.5 EpCAM aptamer results in targeted cellular uptake

**FIGURE 6:**
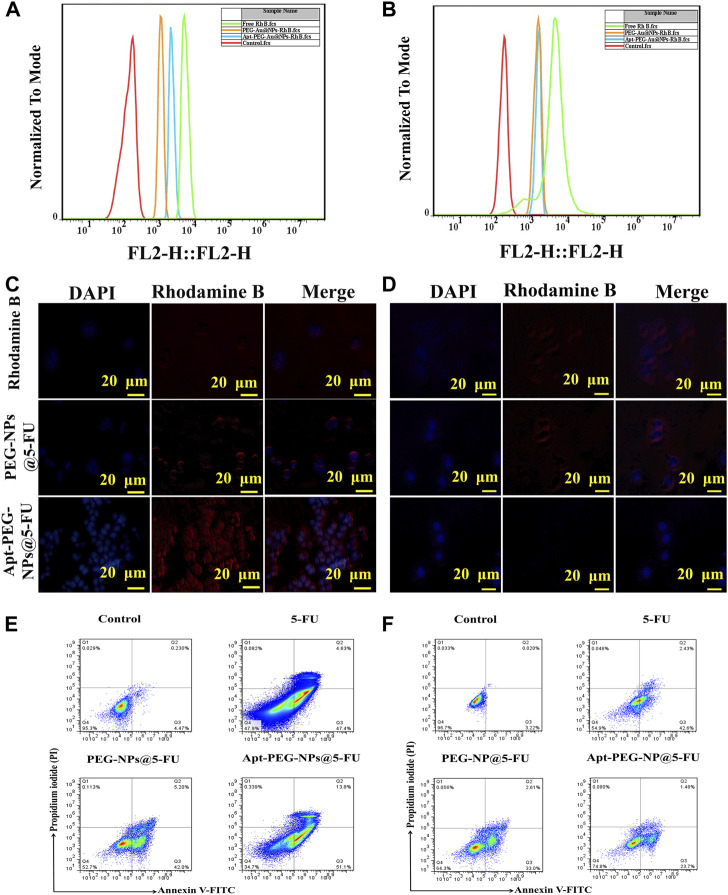
Investigating targeted cellular uptake and cell death mechanism. **(A)** and **(B)** Cellular uptake of targeted and non-targeted NPs in HT-29 and NIH/3T3 cell lines, respectively. Fluorescent microscopy images of Rhodamine B, targeted and non-targeted NPs in **(C)** HT-29 and **(D)** NIH/3T3 cells. DAPI was used to stain the nuclei. **(E)** HT-29 and **(F)** NIH/3T3 cells were treated with free 5-FU, PEG-Au-NPs@5-FU and Apt-PEG-Au-NPs@5-FU and apoptosis was evaluated by annexin V and PI staining using flow cytometry. Abbreviations: *5-FU* 5-Fluorouracil, *Au-NPs* gold nanoparticles, *PEG* Polyethylene glycol, *NP* nanoparticle, *Apt* Aptamer, *EpCAM* Epithelial cell adhesion molecule, *HT-29 cells* Human colorectal adenocarcinoma cells, *NIH/3T3 cells* Mouse fibroblast cell line.

To investigate the ability of aptamer conjugated nanocarriers, bearing a targeting ligand against EpCAM, to deliver the anti-cancer drugs, HT-29 (EpCAM^+^) and NIH/3T3 (EpCAM^−^) cells were treated with PEG-Au-NPs@5-FU, Apt-PEG-Au-NPs@5-FU and free 5-FU and monitored by flow cytometry and fluorescence microscopy. Tracking the cellular uptake by flow cytometry technique illustrated more efficient and higher uptake of targeted nanocarriers in HT-29 cells compared with non-targeted formula. The results indicate that the EpCAM receptor on the surface of HT-29 cells, increased the cellular uptake of Apt-PEG-Au-NPs@5-FU in comparison with PEG-Au-NPs@5-FU (Figures 6A, B), while the fluorescence intensity of NIH/3T3 cells incubated with targeted and non-targeted nanocarriers was identical and no significant difference could be observed. Moreover, fluorescent images of HT-29 and NIH/3T3 cells (Figures 6C, D) further confirmed the specificity of the EpCAM aptamer for targeting HT-29 colorectal cancer cells.

### 3.6 Quantification of apoptotic cell death in NPs-treated cells

Flow cytometry was used for evaluation of cell death mechanism triggered by the non-targeted and targeted NPs in HT-29 and NIH/3T3 cells. In this regard, the annexin V-FITC/PI double staining was carried out to make a distinction between apoptotic and necrotic cells induced by mentioned formulas. The percentages of apoptotic HT-29 cells induced by free 5-FU, PEG-Au-NPs@5-FU and Apt-PEG-Au-NPs@5-FU were calculated as 52.03%, 47.2% and 64.9%, respectively, while for NIH/3T3 cells, they were indicated as 45.03%, 35.61% and 25.18%. The notable increase in apoptotic cell population after treatment with the targeted compound is in agreement with higher receptor mediated cellular uptake and demonstrates more anticancer activity in EpCAM^+^ HT-29 cells in comparison with the EpCAM^−^ cells (Figures 6E, F).

### 3.7 Apt-PEG-Au-NPs@5-FU showed superior anti-tumor efficacy *in vivo*

**FIGURE 7:**
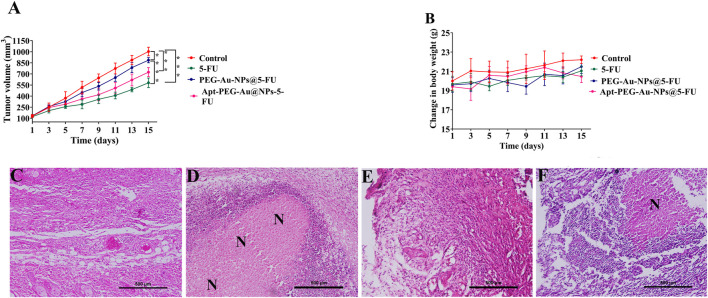
Antitumor efficacy of targeted and non-targeted nanoparticles in mice bearing HT-29 tumors **(A)** Changes in tumor volumes in mice treated with free 5-FU, PEG-Au-NPs@5-FU and Apt-PEG-Au-NPs@5-FU in comparison with control group. **(B)** Body weight changes of mice bearing HT-29 tumors during 15 days of treatment. H&E staining of tumor sections removed from the sacrificed mice in **(C)** control, **(D)** free 5-FU, **(E)** PEG-Au-NPs@5-FU and **(F)** Apt-PEG-Au-NPs@5-FU treated groups. Numbers are expressed as mean ± standard deviation (n = 4, *p < 0.05, ***p < 0.001). “N” represents necrotic area; scale bar: 500 µm. Abbreviations: *Au-NPs* gold nanoparticles, *PEG* Polyethylene glycol, *NP* nanoparticle, *Apt* Aptamer, *5-FU* 5-Fluorouracil, *H&E* Hematoxylin and eosin.

**FIGURE 8:**
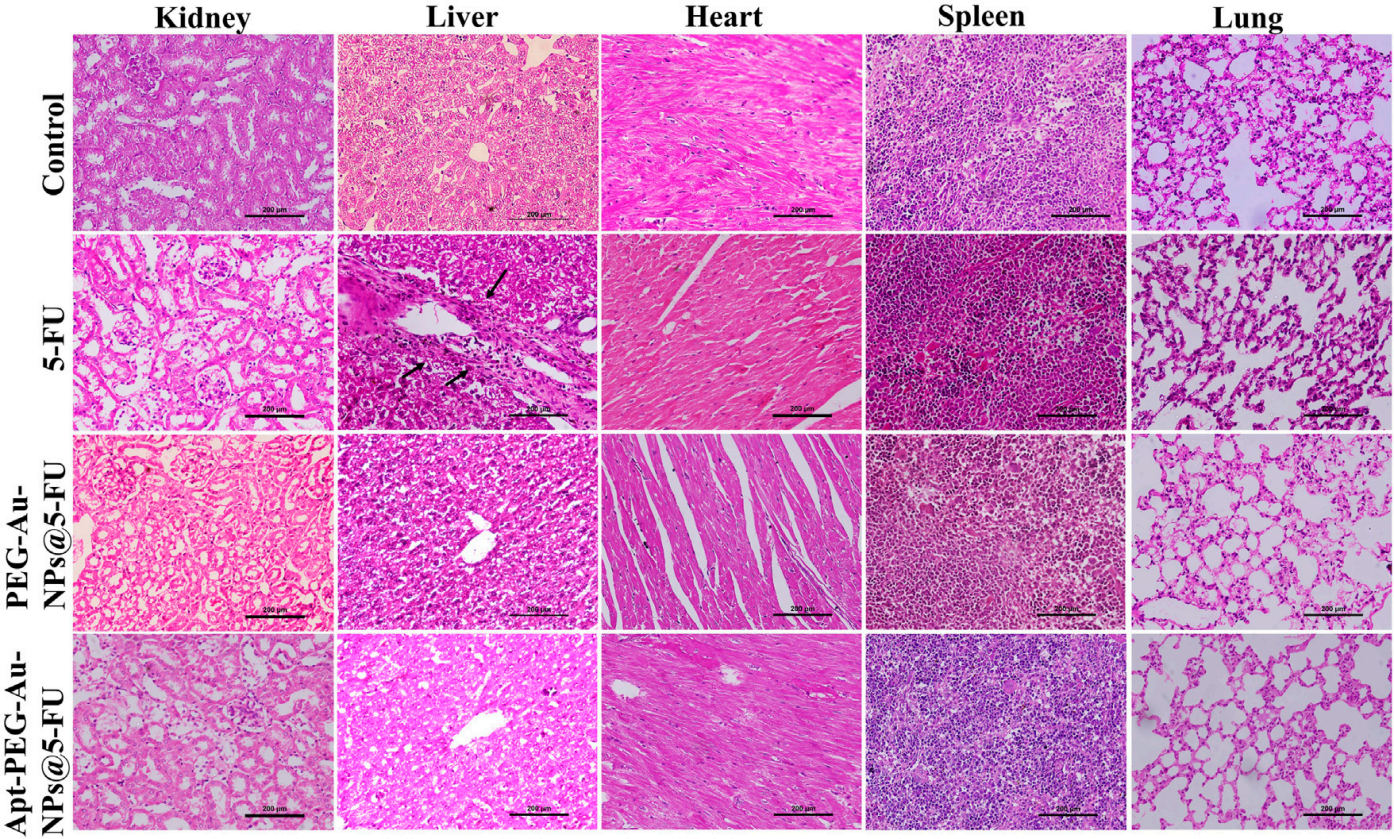
Preliminary systemic toxicity estimation of Apt-PEG-Au-NPs@5-FU in mice bearing HT-29 tumors. Micrographs represent H&E staining of major organs, collected 15 days after treatment. Scale bar: 200 μm Abbreviations: *Au-NPs* gold nanoparticles, *PEG* Polyethylene glycol, *NP* nanoparticle, *Apt* Aptamer, 5-FU 5-Fluorouracil, *H&E* Hematoxylin and eosin.

To evaluate the possible anti-tumorigenic effects of synthetic nanocarriers, we used a xenograft C57BL/6 mouse tumor model. As shown in Figure 7A, although tumor volumes constantly increased in case of PBS control group, other treatments were able to suppress tumor growth with various efficacies. In this regard, no significant difference was observed between free 5-FU and Apt-PEG-Au-NPs@5-FU in tumor growth inhibition. Moreover, the non-targeted group showed a significant difference (*p* < 0.001) with free drug, confirming the importance of using aptamer in active targeting (Figure 7A). High CRC selectivity and effectiveness of Apt-PEG-Au-NPs@5-FU was further confirmed by H&E staining of tumor sections. The remarkable antitumor response might be attributed to the targeting ability of EpCAM aptamer, which is present on the surface of NPs and helps the drug to accumulate at tumor site. The targeting moiety could enhance the concentration of 5-FU within the tumor tissues as evidenced by higher degree of tumor necrosis (Figure 7F) compared to non-targeted NPs. Furthermore, targeted compound was highly efficient in suppressing the tumor growth without noticeable side effects in critical organs including kidney, liver, heart, spleen and lung (Figure 8). In contrast, local accumulation of inflammatory cells in the liver (black arrows) was observed in free 5-FU treated group, confirming its severe side effects.

### 3.8 *In vivo* imaging and biodistribution

**FIGURE 9:**
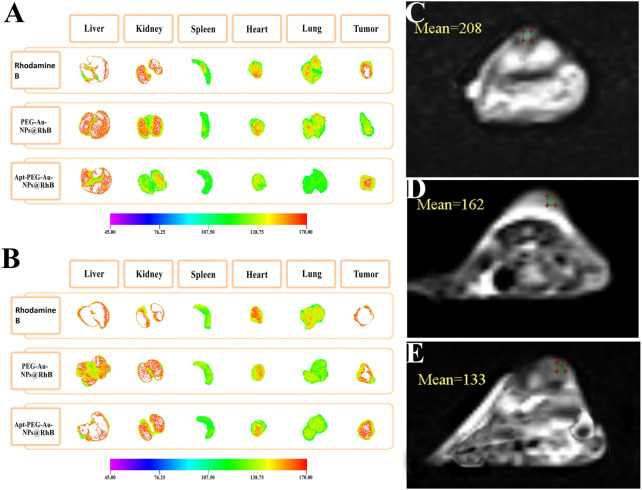
*Ex vivo* fluorescence images of major organs and tumor from mice bearing HT-29 xenograft tumors at **(A)** 12 h and **(B)** 24 h post injection of free Rhodamine B, PEG-Au-NPs@RhB and Apt-PEG-Au-NPs@RhB. MRI scans of the mice implanted with HT-29 cells, at 12 h following various treatments are shown. **(C)** Control, **(D)** PEG-Au-NPs@5-FU and **(E)** Apt-PEG-Au-NPs@5-FU. Abbreviations: *Au-NPs* gold nanoparticles, *PEG* Polyethylene glycol, *NP* nanoparticle, *Apt* Aptamer, *RhB* Rhodamine B.

To assess the distribution and tumor accumulation of the synthesized nanocarriers, mice were intravenously injected *via* tail vein with free Rhodamine B, PEG-Au-NPs@RhB and Apt-PEG-Au-NPs@RhB. The tumor signal intensity of targeted NPs was much stronger than that of non-targeted NPs at tumor tissue during the experimental period (both 12 and 24 h) (Figures 9A, B), further confirming that Apt-PEG-Au-NPs@5-FU can target HT-29 tumors, probably by both passive (EPR effect-mediated) and active (EpCAM aptamer) mechanisms. *Ex vivo* fluorescence signals in major organs indicated that, the amounts of targeted nanocarriers accumulated in the liver, kidney, heart and lung were lower than those of free Rhodamine B and non-targeted NPs after 24 h, suggesting that Apt-PEG-Au-NPs@5-FU could decrease the systemic toxicity of 5-FU in normal tissues.

### 3.9 *In vivo* MRI

MRI scans of C57BL/6 mice bearing HT-29 tumors showed decreasing signal intensity of targeted nanocarriers in comparison with non-targeted ones at 12 h owing to the high concentration of magnetic core within the tumor site (Supplementary Table S1). Our data indicated the high distribution of the Apt-PEG-Au-NPs@5-FU at the tumor region in comparison with PEG-Au-NPs@5-FU at 12 h after the injection (Figures 9C–E).

## 4 Discussion

MSNs have unique physicochemical features that make them ideal multifunctional platforms for drug delivery, imaging, and treatment, a process known as theranostics. One of the major challenges in cancer therapy is selective delivery of therapeutic agents like 5-FU to the tumor site. As a result, utilizing targeted DDSs can help to maximize the therapeutic effects of anti-cancer medications while reducing their negative side effects.

In this work, the anti-tumor properties of targeted magnetic MSNs against CRC cells were examined in comparison to non-targeted NPs. In each step, the synthesized nanocarriers were characterized, and FTIR measurements showed that the functional groups had been successfully attached. The size of SPION-MSNs increased and the zeta potential decreased after adding PEG and aptamer, confirming the production of non-targeted and targeted nanocarriers *via* electrostatic interactions, respectively. The SEM and HR-TEM images of SPION-MSNs demonstrated that the designed nanocarriers were originally spherical, which is consistent with the results reported by Cai et al. (Cai et al., 2019). After drug loading, the specific surface areas and total pore volumes of SPION-MSNs reduced, indicating effective encapsulation and capping in the case of Au-NPs@5-FU. Furthermore, after amination, capping, and PEGylation, the presence of key components such as N, Au, and C indicated the efficiency of these processes, respectively. After PEGylation, the magnetic response of nanocarriers was slightly reduced in comparison with SPION-MSNs. This is due to the successful coating, which reduces the weight ratio of the iron core in the core-shell nanocarriers. TGA data confirmed that the prepared nanocarriers had been successfully modified with NH_2_, Au, PEG, and aptamer. The accurate synthesis and composition of the NPs were confirmed by a step-by-step examination of the generated nanocarriers. Our results are in agreement with other studies that introduced MSNs coated with PEG and polydopamine (PDA) as specific DDSs for CRC therapy (Li et al., 2017; Li et al., 2018). In one study, the size and zeta potential of magnetic MSNs were enhanced from 32.8 nm and −18.5 mV to 89.88 nm and +9.41 mV in their targeted nanostructures (Apt-PEG-NPs) (Sakhtianchi et al., 2019). Applying chemical modifications in each step plays a crucial role in increasing the size of nanocarriers. The increase in nanoparticle diameter could be explained by interactions between new and old functional groups, as well as the development of new chemical bonds. The obtained results are comparable with earlier reports (Kulkarni and Feng, 2013; Behzadi et al., 2017) that show a rise in the size of MSNs following each alteration. Furthermore, Babaei et al. demonstrated that specific capping with Au-NPs was pH responsive for 5-FU release, and that PEG administration resulted in increasing the half-life of the NPs (Babaei et al., 2017). Different studies have shown that NP excretion mechanisms depend on its structure and size. In this context, NPs smaller than 10 nm are swiftly cleared from the kidneys, whereas those larger than 100 nm quickly accumulated in the liver and spleen, leading to a short circulatory half-life (Kulkarni and Feng, 2013; Shiraishi and Yokoyama, 2019). In this study, the targeted formula was about 78 nm, which is more suitable for entrance to tumors and in line with numerous experimental reports as reviewed previously (Behzadi et al., 2017). The successful synthesis of magnetic porous silica NPs (SPION-MSNs), surface modifications and decoration with PEG, and also conjugation of EpCAM aptamer were demonstrated using characterization techniques such as FTIR, zeta potential, SEM, HR-TEM, BET, EDX, VSM, and TGA. Suitability and effectiveness of these nanocarriers were then verified both *in vitro* and *in vivo*.

The release profile of 5-FU from Au-NPs@5-FU comprises two fast and slow phases, as illustrated in Figure 4F, which is consistent with many other studies (Babaei et al., 2017; Iranpour et al., 2021b). Encapsulation of 5-FU within porous silica was intended to prolong the residence time of the drug inside the body and thereby to improve the cellular uptake of the drug by cancer cells. This strategy was important to significantly increase the anti-tumor activity (Lim et al., 2011) and the overall therapeutic efficacy of 5-FU. Furthermore, the release profile indicated a markedly higher 5-FU release rate at pH 5.4 compared with that at physiological pH. During 96 h at pH 7.4, just 1% of 5-FU was released from SPION-MSNs, while at pH 5.4, the cumulative amount of released 5-FU increased to 43%. The presence of gold gatekeepers on the nanocarriers’ pore entrance could explain the pH-responsive sustained-release characteristic. The low pH can function as a stimulant for removing the Au NP gatekeepers from the surface of nanocarriers when the particles are exposed to an acidic environment in tumor cells. As a result, medication release within the targeted tumor cells would benefit from this pH sensitivity. Moreover, prepared nanocarriers not only prevented releasing most of the drug in normal cells, but also guaranteed releasing efficient amounts of the drugs in the target site. Hemolytic activity of SPION-MSNs was determined by measuring the absorption peak of hemoglobin, and compared with PEG-Au-NPs@5-FU. Surface covering with functional PEG resulted in significantly reduced hemolytic activity of nanocarriers, which did not exceed the acceptable hemolysis threshold of 5%. As a result, it can be concluded that PEG-Au-NPs@5-FU are hemocompatible and non-hazardous. Fluorescent microscopy and flow cytometry analyses showed the selective cellular uptake of targeted nanocarriers in HT-29 cells as compared with NIH/3T3 normal cells, which is consistent with other studies. According to Jaimes Aguirre et al. (Jaimes-Aguirre et al., 2017) and Minaei et al. (Minaei et al., 2019), using a folic acid ligand as an active targeting agent could boost the selective uptake of NPs into malignant cells. Overexpression of certain receptors on cancer cells, along with active targeting nanomedicines, could effectively deliver medications into cancer cells *via* receptor-mediated endocytosis (Liu et al., 2020). Moreover, considering the penetration of NPs within the tumor cells is an important factor which can affect the therapeutic responses. In this regard, Kankala et al. decorated Zn-MSN composites with an ultrasmall platinum (Pt) NP in order to enhance the tumor penetration capacity of prepared nanocarriers (Kankala et al., 2020b).

It is generally noticed that the cytotoxic activity of free 5-FU, PEG-Au-NPs@5-FU and Apt-PEG-Au-NPs@5-FU against cancerous and normal cells was time-dependent as reported by other researchers (Nair et al., 2011; Tawfik et al., 2017). As a result, raising the concentration of 5-FU decreased cell viability in both cell types. The targeted nanocarriers showed significantly different impacts on cells that were positive or negative for the EpCAM marker, indicating selective anti-tumor activity of our formula. These findings were supported by apoptosis analysis, which revealed that the targeted formulation caused significantly more cell death in HT-29 cells than in NIH/3T3 cells. Our findings support previous research that found 5-FU-loaded NPs induced apoptosis in colon cancer cells (Jiang et al., 2018; Liu et al., 2018). In addition, the anti-tumor activity of non-targeted and targeted nanocarriers was evaluated in immunocompromised mice bearing HT-29 tumors. In comparison to the non-targeted form and the PBS group, tumor volume was significantly reduced in mice treated with targeted nanocarriers. Quantitative measures revealed that tumor volumes increased fast in the control group, reaching around 1,000 mm^3^, but were limited to 600 mm^3^ in the targeted group. Other studies have also shown that targeted nanocarriers can achieve exceptional tumor suppression (Li et al., 2017; Li et al., 2018; Sun et al., 2019; Xu et al., 2020; Khatami et al., 2021). Furthermore, the body weight of mice injected with specific nanocarriers did not vary significantly, indicating that there were no noticeable side effects. The *in vivo* imaging results of the two animal groups treated with either non-targeted or targeted formulations suggest that the high tumor targeting and no remarkable side effects of Apt-PEG-Au-NPs@5-FU nanocarriers during the treatment period might be due to specific aptamer and EpCAM interactions leading to successful internalization of these nanocarriers. In addition, at 12 h after the injection, the fluorescence intensity of RhB was first discovered in the liver and kidney before appearing at a low level in the tumor site. This result is consistent with the finding that MSNs are mainly metabolized by the liver and kidneys (Maeda, 2001). After 24 h, the fluorescence intensity increased in the tumor for targeted formula. Moreover, there was no significant differences in tumor inhibition between targeted form and free 5-FU, while significant side effects were observed in free 5-FU group, probably due to its low molecular weight leading to non-specific uptake in normal tissues. Similarly, MRI results demonstrated the increased lesion localization and tumor affinity of Apt-PEG-Au-NPs@5-FU in comparison with PEG-Au-NPs@5-FU after 12 h. These results support the notion that targeted nanocarriers can be used as a preferred theranostic platform for effective CRC inhibition. This finding is consistent with the results reported by Alkahtane *et al.* which illustrated that chitosan coated Fe_3_O_4_ nanohybrid enhanced MRI contrast ability for CRC therapy (Alkahtane et al., 2022). Moreover, Zou et al. reported that MUC1‐SPIONs as a targeted construct can effectively improve pancreatic tumor‐targeting as confirmed by both *in vitro* and *in vivo* MRI studies (Zou et al., 2019). The histological imaging of critical organs confirmed the negligible side effects of targeted formula in the kidneys, liver, heart, spleen and lungs along with high eradication of tumor tissue. Our results demonstrated that Apt-PEG-Au-NPs@5-FU could specifically target the tumor tissues, resulting in the enhanced 5-FU delivery and anti-tumor effects. We suggest that the Apt-PEG-Au-NPs@5-FU might be an effective formula for colorectal cancer therapy, however it still requires further investigations before it can reach the clinic. MRI-based diagnostic and therapeutic protocols are being developed with promising clinical value, and will ultimately provide the theoretical and technical foundation for precise diagnosis and efficacious treatment of CRC.

## 5 Conclusion

The EpCAM-conjugated PEGylated SPION-MSNs synthesized in this study proved to be an interesting platform for therapeutic applications. The presence of PEG and aptamer was confirmed by FTIR, zeta potential, VSM and TGA analyses, demonstrating the efficiency of the synthetic route. The PEG layer can increase its systemic circulation, inhibit the removal of NPs by the mononuclear phagocytic system, and alter some physicochemical properties of the NPs, such as stability, and drug loading and release conduct, while the EpCAM moiety is aimed to increase the targeting of nanocarriers for cancer cells. Overall, we developed a targeted and pH-sensitive DDS based on EpCAM Apt-functionalized MSNs, which can successfully deliver 5-FU to CRC cells and enhance anti-cancer activity, while reducing systemic off-target toxicities. These Apt-PEG-Au-NPs@5-FU nanocarriers have several advantages as a therapeutic option in cancer therapy, including 1) high drug loading capacity, 2) enhanced drug release rate at acidic pH, and 3) ability of targeting HT-29 tumors by EpCAM Apt-mediated mechanisms.

## Data Availability

The original contributions presented in the study are included in the article/Supplementary Material, further inquiries can be directed to the corresponding authors.

## Glossary

APTES: Aminopropyltriethoxysilane
BET: Brunauer-Emmett-Teller
BJH: Barrett-Joyner- Halenda
CTAB: Cetyltrimethylammonium bromide
CRC: Colorectal cancer
DAPI: 4’, 6-diamidino-2-phenylindole
DDSs: Drug delivery systems
DMEM: Dulbecco’s modified Eagle’s medium
DLS: Dynamic light scattering
EDX: Energy-dispersive X-ray spectroscopy
EE: Encapsulation efficiency
EpCAM: Epithelial cell adhesion molecule
FBS: Fetal bovine serum
FITC: Fluorescein isothiocyanate
FTIR: Fourier transform infrared
5-FU: 5-Fluorouracil
H&E: Hematoxylin and eosin
HR-TEM: High resolution transmission electron microscopy
LC: Loading capacity
MRI: Magnetic resonance imaging
MTT: 3-(4, 5-dimethylthiazol-2-yl)-2, 5-diphenyltetrazolium bromide
MSNs: Mesoporous silica nanoparticles
NH_4_OH: Ammonium hydroxide
NPs: Nanoparticles
OD: Optical density
PDI: Polydispersity index
PEG: Polyethylene glycol
PI: Propidium iodide
RBCs: Red blood cells
RPMI 1640: Roswell Park Memorial Institute 1640
SEM: Scanning electron microscope
SPIONs: Superparamagnetic iron oxide nanoparticles
TEOS: Tetraethyl orthosilicate
TGA: Thermogravimetric analysis
VSM: Vibrating-sample magnetometer
