# Supplementary material for: Using magnetic mesoporous silica nanoparticles armed with EpCAM aptamer as an efficient platform for specific delivery of 5-fluorouracil to colorectal cancer cells
Source: Front Bioeng Biotechnol. 2023 Jan 6;10:1095837. doi: 10.3389/fbioe.2022.1095837 (PMC9853966; doi:10.3389/fbioe.2022.1095837)
Supplement: Supplementary file 1 [file DataSheet1.docx]

**Supplementary Materials**

**Using magnetic mesoporous silica nanoparticles armed with EpCAM aptamer as an efficient platform for specific delivery of 5-fluorouracil to colorectal cancer cells**

Aseel Kamil Mohammad Al-Mosawi^1^, Ahmad Reza Bahrami^1,2^, Sirous Nekooei^3^, Amir Sh. Saljooghi^4,5*^, Maryam M. Matin^1,5,6*^

^1^Department of Biology, Faculty of Science, Ferdowsi University of Mashhad, Mashhad, Iran

^2^Industrial Biotechnology Research Group, Institute of Biotechnology, Ferdowsi University of Mashhad, Mashhad, Iran

^3^Department of Radiology, Qaem Hospital, Mashhad University of Medical Sciences, Mashhad, Iran

^4^Department of Chemistry, Faculty of Science, Ferdowsi University of Mashhad, Mashhad, Iran

^5^Novel Diagnostics and Therapeutics Research Group, Institute of Biotechnology, Ferdowsi University of Mashhad, Mashhad, Iran

^6^Stem Cells and Regenerative Medicine Research Group, Academic Center for Education, Culture and Research (ACECR)-Khorasan Razavi, Mashhad, Iran

^*^co-corresponding authors: [matin@um.ac.ir](mailto:matin@um.ac.ir) and saljooghi@um.ac.ir


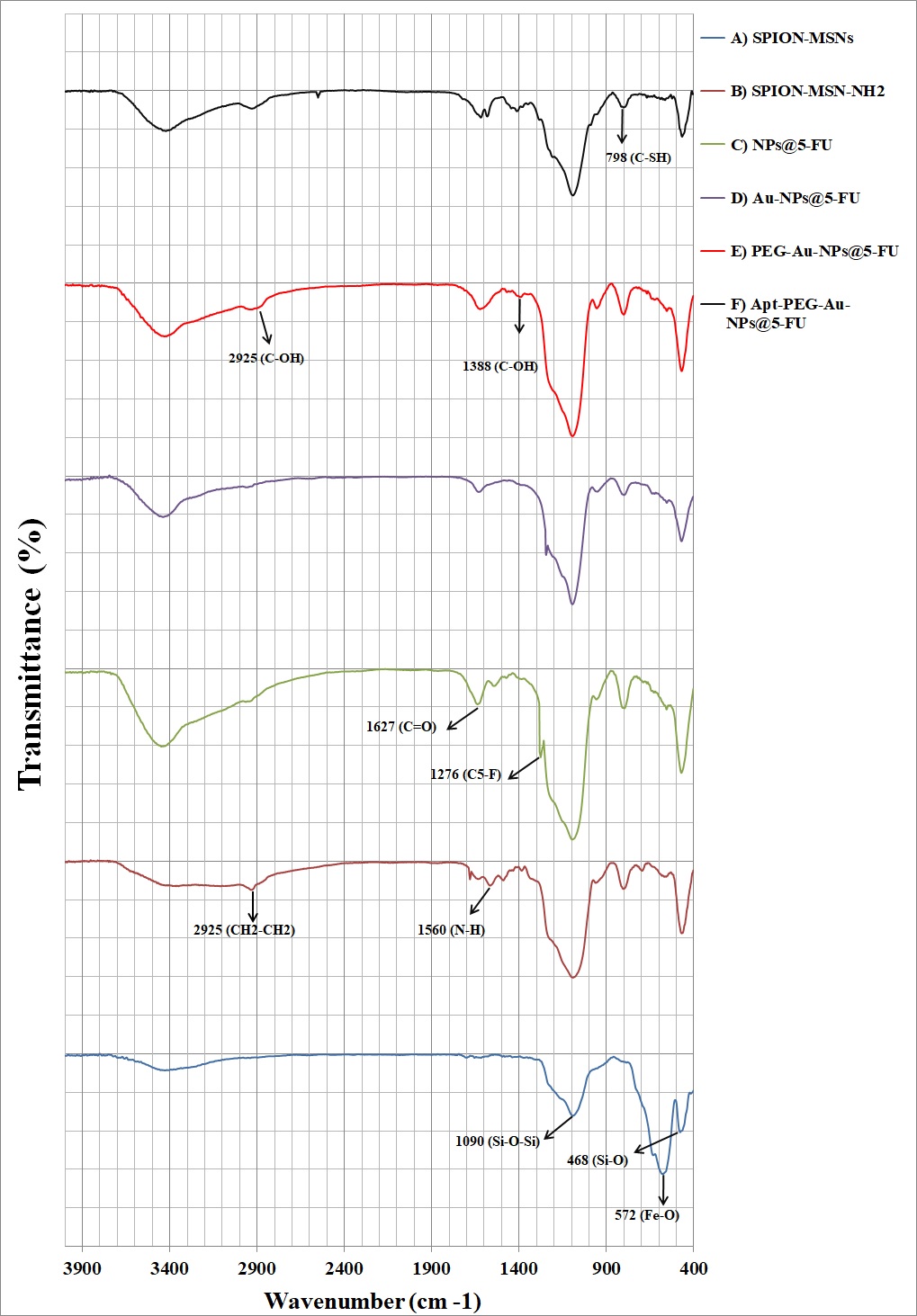


Fig. S1: Fourier transform infrared (FTIR) spectra of synthetic nanocarriers in each step of the fabrication.

Table S1: Signal intensity of PEG-Au-NPs@5-FU and Apt-PEG-Au-NPs@5-FU after 12 and 24 h post injection as revealed by MRI.

| Signal intensity (AU) | 12 h |
| --- | --- |
| control | 100 |
| PEG-Au-NPs@5-FU | 75 |
| Apt-PEG-Au-NPs@5-FU | 61 |
